# Editorial: Community series in recent advances in potential biomarkers for rheumatic diseases and in cell-based therapies in the management of inflammatory rheumatic diseases, volume III

**DOI:** 10.3389/fimmu.2025.1771049

**Published:** 2026-01-06

**Authors:** Eric Toussirot, Katarzyna Bogunia-Kubik, Philippe Saas

**Affiliations:** 1 Université Marie et Louis Pasteur, Centre Investigation Clinique INSERM CIC-1431, CHU de Besançon, Besançon, France; 2 Hirszfeld Institute of Immunology and Experimental Therapy, Polish Academy of Sciences, Wroclaw, Poland; 3 Institute for Advanced Biosciences, Inserm U1209, CNRS UMR 5309, Université Grenoble Alpes, Grenoble, France; 4 Etablissement Français du Sang Auvergne-Rhône-Alpes, Grenoble, France

**Keywords:** April, cell death, chemokines, interferon, non-coding RNA

This Research Topic focuses on recent advances in the description of biomarkers for the diagnosis, prognosis and the disease activity (or severity) of inflammatory rheumatic diseases (IRDs) or of systemic autoimmune diseases, as well as on recent advances in cell-based therapies for the treatment of these conditions. This is the third volume on this Research Topic (Saas et al., Bogunia-Kubik). It includes 7 articles with 4 original articles and 3 reviews of the literature, mainly focusing on biomarkers. They described new biomarkers in IRD and they can be subdivided as follows:

1. biomarkers for treatment response,
2. biomarkers for disease progression or for diagnosis of specific organ involvement,
3. identification of biomarkers that could represent a new therapeutic target,
4. biomarkers for cell death mechanisms.

The IRDs or systemic autoimmune diseases that are specifically involved in this Research Topic are rheumatoid arthritis (RA), juvenile idiopathic arthritis (JIA), systemic sclerosis (SSc), systemic lupus erythematosus (SLE) and Sjögren’s disease (SD). Thus, new valuable information in the field of biomarkers in IRD/systemic autoimmune diseases are provided in this volume III of the Research Topic.

## 1 Identification of novel biomarkers for treatment response in Sjögren disease

Sjögren disease is a chronic systemic disease characterized by exocrine gland dysfunction and also extra-glandular manifestations. The pathophysiology of this disease remains partially understood with a clear involvement of B cell autoimmunity. Type I interferon is considered as a key driver of the disease while type II interferon may potentially play a role. Despite the current lack of specific targeted therapies in SD, conventional treatments such as hydroxychloroquine (HCQ) or leflunomide (LEF) are proposed and included in the pharmacological treatment options of the disease (1). Wong et al. evaluated biomarkers for treatment response and disease activity in SD by means of a proteomic approach. They described a set of proteins differentially expressed between patients with SD and healthy controls, with a down regulation during HCQ and LEF treatment. Two baseline proteins belonging to the chemokine family (CXCL10 and CXCL11) were specifically identified as biomarkers for treatment response. They also determined that type II IFN signatures were observed in the monocytes of patients and were associated with improvement in disease activity, supporting a role for a type II IFN associated immune response in SD.

## 2 Biomarker for disease progression or for diagnostic of organ damage

Juvenile idiopathic arthritis (JIA) is an IRD affecting pediatric subjects with different clinical and biological presentations (2). Oligoarthritis is the most common form that can progress toward a more severe polyarticular presentation with a potential erosive course. Predictive markers for the progression from an oligoarticular to a polyarticular presentation are lacking. In their study, Raggi et al. evaluated new onset oligoarthritis JIA in order to identify biomarkers for progression to a polyarticular disease. They analyzed synovial fluid (SF) and peripheral blood (PB) samples of their patients by evaluating immune cell subsets (e.g., T lymphocytes, CD4^+^ and CD8^+^ T cells and their activation/maturation state, monocytes/macrophages) in PB using flow cytometry, and expression levels of 37 different surface markers of extracellular vesicles (EV) in SF. They identified novel immunological biomarker panels combining the analysis of circulating immune cell subsets with SF EV markers. For SF, the authors identified as biomarkers the CD3:CD14 cell ratio and the expression levels of HLA-ABC and CD3 on EV. For PB, they selected the following markers: the percentage of HLA-DR^+^ T cells, proportions of naive and effector memory (EM) T cell subsets (based on CD45RO and CCR7 expression), and the EM:naive ratio within both CD4^+^ and CD8^+^ T cell subsets. This combination of PB and SF biomarkers can stratify at baseline patients who will progress to a polyarticular disease.

Interstitial lung disease (ILD) is a common and severe extra-articular manifestation of systemic autoimmune diseases that can lead to progressive lung function alteration. This organ involvement is shared by various IRD and systemic autoimmune diseases, and represents a serious concern in SSc. There is an unmet need for biomarkers and clinical/biological or imaging tools for the diagnosis and prediction of ILD in systemic autoimmune diseases. Dai et al. specifically examined this relevant question in patients with various systemic autoimmune diseases including SSc. They performed a transcriptomic and bioinformatics analysis to identify upregulated genes in lung fibroblasts of SSc-associated ILD in publicly available databases. Then, they analyzed PB, bronchoalveolar lavage fluid (BALF) and lung tissue samples from patients with systemic autoimmune disease with or without ILD and healthy controls. They can identify one protein upregulated in the lung tissue of ILD patients, microfibril-associated protein 5 (MFAP5). MFAP5 concentrations were found elevated in the serum and BALF of the patients with ILD compared to the patients without ILD or to healthy controls. The concentrations of that protein also correlated with the extent of ILD. Finally, MFAP5 was also found elevated in a mouse IDL model (*i.e*., bleomycin-induced lung fibrosis). Consequently, these results suggest that MFAP5 could be a marker for diagnosing and predicting ILD in systemic autoimmune diseases. This works fits with the identification by single-cell RNA-sequencing of a MFAP5^high^ fibroblast subset in lung tissues of patients with SSc-associated ILD (3).

## 3 Identification of biomarkers that could represent a new therapeutic target

Systemic autoimmune diseases are characterized by abnormal activation, proliferation and survival of B cells with the production of autoantibodies, for instance anti-citrullinated peptide antibodies in RA. Two critical B cell activating factors are described, proliferation-inducing ligand (APRIL) and B-cell activating factor (BAFF), playing essential roles in the regulation of B cell maturation and antibody production. BAFF has been identified has a key factor in certain systemic autoimmune diseases such as SLE and SD, leading to the development of the specific biological agents belimumab in SLE (4) and ianalumab in SD (5). In their review article, Poznyack et al. provided a full and complete overview of the current knowledge of APRIL and its role in autoimmunity, mainly in RA, SLE and SD, highlighting future opportunities for targeting this molecule in the treatment of systemic autoimmune diseases. Specific APRIL antagonists are discussed.

The Nuclear Factor Kappa B (NF-κB) transcription factor family regulates several genes involved in inflammatory responses. The NF-κB pathway is deregulated in RA and participates to RA pathophysiology, leading to chronic inflammation and joint destruction. Non-coding RNA (ncRNA) are involved in the control of gene expression and biological responses. Different ncRNA have been described, including microRNAs, long non-coding RNAs and circular RNAs. Seyedi et al. reviewed ncRNAs associated with the NF-κB signaling pathway in RA. This review analyzed how ncRNAs could be used as potential early detection markers and therapeutic targets, highlighting their potential therapeutic interest in RA.

## 4 Biomarkers for cell death mechanisms

Programmed cell death (PCD), including apoptosis, autophagic cell death, pyroptosis, necroptosis and ferroptosis corresponds to different biological processes that are involved in tissue balance and regulation. One may distinguish immunologically silent PCD (*i.e*., apoptosis) from pro-inflammatory PCD pathways (e.g., pyroptosis, necroptosis, or ferroptosis), these latter being associated with the plasma membrane rupture and the release of pro-inflammatory damage-associated molecular patterns in the extracellular milieu (6). Alteration in PCD pathways has been implicated in the pathophysiology of autoimmune diseases, including RA (7) and administration of apoptotic leukocytes has been proposed to treat RA (Toussirot et al.). An imbalance in PCD can lead to the release of these inflammatory mediators that can induce chronic synovial inflammation, bone and joint damage. Programmed cell death may also be regulated by ncRNA. The review by Wen et al. analyzes the regulation of cell death in RA, its implication in RA pathophysiology and also the crosstalk with ncRNA. They also reported how traditional Chinese medicine formulations may modulate these cell death networks, providing novel insights for a targeted RA treatment.

Cuproptosis is another type of pro-inflammatory cell death that has been implicated in the development of RA. In their original article, Li et al. examined the expression and biological function of cuproptosis-related genes (CRG) in RA. Ten differentially expressed CRG between patients with RA and controls were described as well as their functional roles and potential interest as a therapeutic target in RA.

Overall, this third volume of the Research Topic usefully completes the two previous volumes (Saas et al., Bogunia-Kubik) and has been very stimulating for the handling editors. Using various experimental approaches, it provides a comprehensive perspective on new biomarkers in the field of diagnostic, prediction and treatment outcome of IRDs and systemic autoimmune diseases.
